# Organ donation: a cultural and religious vision. The Barcelona project

**DOI:** 10.3389/fpubh.2026.1773397

**Published:** 2026-04-08

**Authors:** Jorge Twose, Agustí Iglesias, David Paredes-Zapata, Gurch Randhawa, Emma Arcos, Jaume Tort

**Affiliations:** 1 Organització Catalana de Trasplantaments, Barcelona, Spain; 2 Generalitat de Catalunya Department de Justícia, Barcelona, Spain; 3 Hospital Clinic de Barcelona, Barcelona, Spain; 4 Institute for Health Research, University of Bedfordshire, Luton, United Kingdom

**Keywords:** acknowledgement, cultural, family consent, family refusals, informative materials, organ donation, religion, transplantation

## Abstract

Trust and credibility in donation is a key factor for the Spanish model. In 2018, we began in Barcelona the Barcelona Reflection Project: Organ Donation, a Cultural and Religious Perspective project as a multidisciplinary approach to discuss donation and transplantation concepts with religious leaders from Catalonia. The aim was to enhance understanding of religious and cultural aspects that could encourage dialogue and reduce refusal rates for donation. The project comprised seven activities: two open conferences and five closed seminars. Each workshop focused on a particular faith: Catholicism, non-Catholic Christianity, Islam, Judaism, and a mixture of Asian religions. In total, 70 religious or cultural leaders, 11 Transplant Coordinators, and 5 religious diversity experts participated. The workshops confirmed that none of the major religions opposes organ and tissue donation, but discussion of donation procedures and funeral aspects were not common within the communities and can influence donation acceptance by relatives. To improve public knowledge and facilitate community dialogue, we developed informative materials for donors and families, but also for health professionals. The Barcelona project facilitates an inclusive multidisciplinary forum for all religions, creating a shared space to exchange knowledge and foster productive dialogue and cooperation. The percentage of consent for organ donation in Catalonia over the past 10 years stands at 81.8% with statistically differences according to the origin and year.

## Introduction

The success of the Spanish transplant model (1) is multi-factorial, linked to a well stablished legal framework, a public health system with universal coverage, coordination at three levels—national, regional and hospitals—the integral role of the Transplant Coordinators (TC), and a medical consensus between health teams, authorities and scientific community (2, 3). All of this, supported by a positive attitude and trust relationship in favour of organ donation (4, 5) of the Spanish society.

The hospital-based donation process comprises several distinct phases, all coordinated by the TC or the coordination team. These professionals, most of whom in Spain are intensive care physicians, are responsible—together with medical and nursing staff from the hospital—for managing each stage of the process. Their tasks include identifying potential donors, maintaining physiological stability, assessing organ viability, providing support during the grieving process, maintaining open-dialogue with the families of potential donors, and coordinating organ retrieval teams in the operating theatre. The purpose of open-dialogue with the family is to provide clear and understandable information, address any questions, and ensure that the donation decision is made freely and knowingly within a context of grief. This conversation is grounded in active listening, empathy and emotional support, always prioritising the family’s well-being and respect for the donor’s values and wishes.

The Spanish donation law is an opt*-*out system (presumed consent), that means everyone is a donor unless they have expressed otherwise during their lifetime (6). However, in real-life circumstances, the TC seeks evidence of the deceased’s willingness to donate and always seeks consent to donation from the family via open-dialogue. Therefore, within Spanish society, trust and credibility in the transplant system is essential. To this end, far from large and expensive public campaigns, the focus from the outset was on bringing donation closer to families through the training and expertise of TC (7). The creation of this professional role, along with facilitating close collaboration with the media, has been key elements in raising positive awareness in Spanish society.

The percentage of family refusals is a strategic indicator of the donation and transplantation system. Despite being in a better situation than most neighbouring countries (8, 9), Spain and in particular in Catalonia region it has seen a slight upward trend in family refusals in recent years. We believe this is due to the influence of multiple factors, some of them related to deep changes in our societies after the pandemic; new donation processes such as pre-donation interviews in cases of intensive care oriented toward donation (ICOD) (10); the persistence of myths and taboos related to donation, and the increasing cultural and religious diversity of our society (11).

Unfortunately, deceased donation is not yet a consolidated reality in some areas of the world, as shown by the annual reports of international registries such as the WHO’s Global Observatory of Donation and Transplantation (GODT) (12) or the International Registry on Donation and Transplantation (IRoDAT) (13). An example of this fact, is the world map of actual deceased organ donors per million of population rate, published by GODT (14). Spain and the United States have the highest donation rates worldwide, followed by European countries such as Portugal, France or Italy. At the other end of the spectrum are Asian countries (where living donation predominates) and most of Central and South America countries. Few African countries report any type of donation, mainly living.

To promote organ and tissue donation among culturally and religiously diverse communities, the Catalan Transplant Organization (OCATT) launched the Barcelona Reflection Project: Organ Donation, a Cultural and Religious Perspective (2018–2019), with support from the Catalan Directorate General for Religious Affairs (DGRA) and Palau Macaya (“la Caixa” Foundation). Its goals were to foster dialogue with community leaders and to improve health professionals’ understanding of how religious beliefs influence attitudes toward donation.

Six years after the projects closure (June 2019), this study aims to share the knowledge gained, review the educational materials for both the public and health professionals in particular TC and present the evolution of donation consent rates over these last years.

## Context

Catalonia is a northeast region of Spain with 8.0 million of inhabitants. In 2025, the generosity and altruism of 370 deceased organ donors and their families, a rate of 46 donors per million of population (pmp), 174 renal living donors and the solidarity of other Spanish regions, enabled 1,356 organ transplants in 2025 (15) (Rate: 167 transplants pmp), probably one of the highest in the world (12). The donation rate in Spain is even higher, 52 pmp donors (2,547 deceased donors) led to 6,334 transplants in 2025 (16) (Rate: 129 transplants pmp).

In Catalonia region, cultural and religion reasons are, in some cases, explanation for family refusals to donation (5–15% of family refusals (15)). Although, none of the major religions are against donation we have seen (17), similar to other authors (18, 19), significant differences in terms of lower of donation in people originally born from North Africa and sub-Saharan Africa, the Middle East and Asia. We believe these results do not reflect a lack of solidarity or generosity within these communities but rather stem from insufficient or inadequate information and a certain degree of mistrust in their own health system that is transferred to the Spanish one. A recent Catalan Health Survey (ESCA) (20), showed a lower predisposition to be a donor or to donate organs from a relative member in citizens born in African (only 20–50% in favour of donation) and Asian countries (only 16–38% in favour of donation). The integration of new population groups, because of migratory movements, into the traditions, behaviour, and realities of the host countries requires time, effort, interest and motivation, which lead to improved levels of trust (4, 21).

### Reflection project: organ donation, a cultural and religious perspective

**Figure 1:**
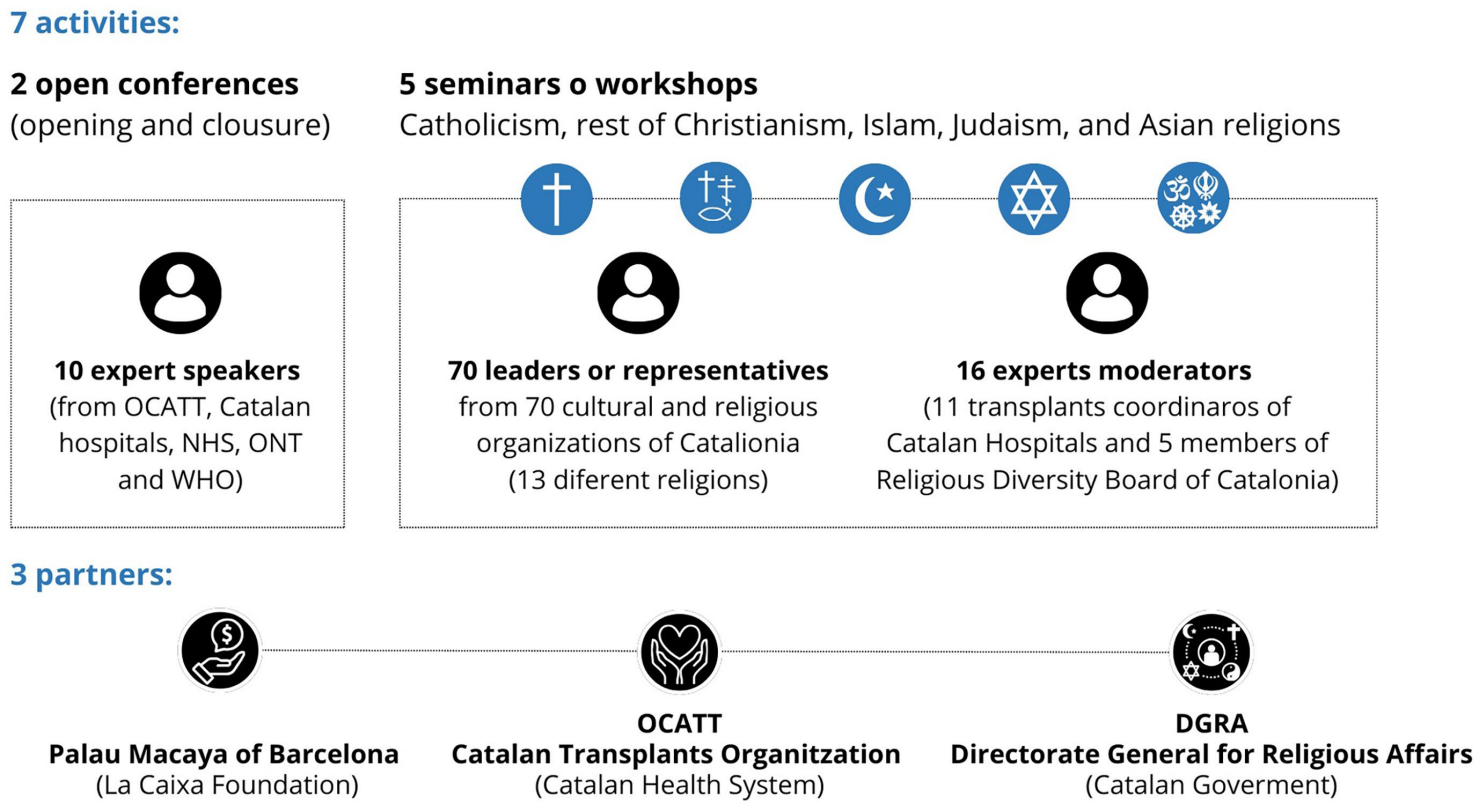
The Barcelona (religious and donation) reflection project. The project took place at the Palau Macaya in Barcelona between 2018 and 2019, consisting of 7 in-person events: 2 open conferences and 5 closed seminars. Each workshop focused on a particular faith or group of religions: Catholicism, rest of Christianity, Islam, Judaism, and Asian religions (mainly Hinduism, Sikhism, and Buddhism).

The Barcelona Reflection Cycle on donation and religious perspective emerged from a competitive call by Palau Macaya (*“la Caixa”* Foundation), aimed at generating new knowledge across sectors of society through public-private collaboration. To this end, in addition to the financial contribution (10,000€), project teams were required to design a program that combined open and closed activities (maximum seven activities) to encourage debate and the exchange of ideas among participants, utilizing the spaces of the historic Palau Macaya building in Barcelona. OCATT, together with the DGRA in Catalonia, designed a program (22) with seven in-person sessions (Figure 1): two public conferences, opening and closure, and five closed seminars tailored to different religious traditions: Catholicism, Non-Catholic Christianism, Islam, Judaism and, finally, a mixture of oriental religions.

The cycle began in September 2018, with the inaugural conference *“Donation and transplantation in our environment, a model of success that does not stop.”* National and international speakers framed the project in the current context, explained the key aspects of donation and transplantation and showed a previous experience on donation and cultural and religious diversity. The objective of the session was to explain the situation of cultural diversity in Catalonia, the basic concepts of donation, the donation process and the key figure of the hospital transplant coordinator, but also, the Spanish transplant model and the socio-cultural aspects that may condition it. In addition, we had the opportunity to learn about the United Kingdom experience on the treatment of cultural and religious diversity in relation to donation.

The five specific workshops (Catholicism, Non-Catholic Christianism, Islam, Judaism and oriental religions) were organized inviting representatives of cultural and religious entities in Catalonia related with each seminar. Overall, 70 different representatives of numerous entities linked to Catholicism and non-Catholic Christianism (Evangelists, Mormons, Jehovah’s Witness, Orthodox and Adventists), Islam, Judaism, Buddhism, Hinduism, Taoism, Sikhism or the Baha’i Faith, accompanied us during all the sessions. The number and the name of entities who participated in each of the five workshops are available in the project webpage (22). Throughout these seminars, it was possible to delve deeper and learn more about those aspects that could influence a favourable attitude towards organ and tissue donation or, on the opposite, could represent barriers to be taken into account. They were enriching, interesting and productive seminars thanks to the good climate of debate established that allowed for open, friendly and direct reflection. Eleven hospital transplant coordinators, together with five members of the Advisory Council for Religious Diversity of the Government of Catalonia, moderated the seminars.

The conference *“Building trust and knowledge: Organ donation from a cultural and religious perspective,”* held on January 2019, closed the reflection cycle at the Palau Macaya. If the opening conference aimed to explain the conceptual framework of the project, on this occasion, the objective was much more practical. It was about explaining the development of the seminars, showed the preliminary results derived from the debates as well as the main conclusions of the cycle. In addition, professional experts from national (Organizacion Nacional de Trasplantes, ONT and OCATT) and international health organizations (World Health Organization, WHO) assessed the implications of the project and showed the situation of transplantation in the world.

The initiative gathered over 90 participants from more than 70 entities representing all 13 faiths in Catalonia (22).

### Evaluation of the percentage of family consent to donation

A critical step in the donation process is the family open-dialogue led by TC to assess the potential donor’s willingness to donate. Always, in all cases without exception, healthcare professionals hold this meeting with the relatives of the potential donor, with the aim of understanding the deceased person’s wiliness to become a donor. If the individual did not clearly express their wishes during life, the family must confirm the absence of known objections and provide consent for organ and tissue donation.

The percentage of family refusals refers to the proportion of family dialogues that do not result in donation due to lack of consent. This information is systematically recorded in the donation and transplant registry of Catalonia, where reporting is mandatory.

Place of birth serves as a proxy for cultural or religious background. For this analysis, we categorized origin into 10 groups: Spain, rest of Europe, North Africa, sub-Saharan Africa, the Middle East, Asia, North America, Central and South America, and Oceania. We performed all the analyses using the statistical package STATA.

## Detail

### Reflections on the different religious groups participating in the five seminars of the cycle

A summary of the main reflections obtained from the different seminars, are also available in the webpage (22):

In Christianity, organ and tissue donation is broadly accepted, particularly within the Catholic Church, which explicitly supports it—endorsed by Popes John Paul II, Benedict XVI, and Francis. Other Christian denominations, such as Protestant and Orthodox traditions, generally do not oppose donation but lack a unified stance due to decentralised leadership. In these cases, donation is often framed as a matter of individual conscience, leaving the decision to each believer.

Islamic doctrine strongly emphasises charity and the duty to save lives—an act considered among the highest virtues in Islam, as reflected in the Quranic verse: *“Whoever saves a life saves all of humanity.”* While there is broad consensus that Islam does not forbid organ donation and many religious leaders support it, the absence of a unified authority and the diversity of interpretations across countries, traditions and world regions complicate the establishment of a clear doctrinal position. Living donation is widely accepted, whereas cadaveric donation can raise more concerns, particularly with funeral rites, in special in the country of origin of the donor, when body should be returned to their original country for been buried. Promoting structured dialogue between healthcare professionals and Muslim leaders is essential to overcome mistrust and misinformation.

In Judaism, the principle of *pikuach nefesh—*the preservation of human life—supersedes most religious precepts. The Torah, echoing similar teachings in Islam, states that saving one life is akin to saving all of humanity. There is broad consensus that Judaism does not prohibit organ donation, and prominent authorities, such as the Chief Rabbi of Israel, have endorsed it. However, certain funeral practices, such as ritual washing, immediate burial, and restrictions on handling the body during the Sabbath or major holidays, may present challenges. In our experience, ultra-Orthodox communities tend to be more hesitant and, in our Project, were among the few who declined to participate in the discussions.

Asian religions present certain challenges to organ donation, often linked to beliefs surrounding the dying process. In Buddhism, for instance, the bardo state—a 72-h post-mortem period during which the body should not be disturbed—can hinder donation. However, core values such as compassion (Buddhism), love (Hinduism), and service (Sikhism) align closely with the altruistic nature of donation. The absence of explicit sacred texts and the diversity of beliefs and authorities complicate the establishment of a unified doctrinal stance. Nevertheless, respected figures like the Dalai Lama and the World Hindu Council have publicly supported donation. In practice, decisions are often guided by individual conscience.

The Baha’i Faith does not consider anything in its doctrine against donation, provided the body is treated with respect and justice is applied in the distribution or allocation of the obtained organs and tissues. In fact, it promotes harmony between science and religion, advocates constant dialogue in decision-making, and avoids conflict between the two.

### Informative materials derived from the cycle

Several findings in this paper stem from discussions held during the five seminars of the Barcelona Reflection Cycle. These insights resulted in the development of a series of multilingual leaflets on organ donation from the perspective of six major religions, designed for families from our diverse communities (22). OCATT and the DGRA created these materials with input from all participating organizations. Before publication, every collaborating entity—including some that initially declined participation—reviewed and approved the final texts.

In June 2022, we launched the Guide on Religious Diversity and Organ and Tissue Donation (23), aimed at helping transplant coordinators better engage with families from different faith backgrounds. The guide outlines doctrinal arguments supporting donation, potential concerns or limitations, and considerations regarding the handling of the body across 13 religious traditions. It also includes contact information and relevant resources for healthcare professionals. As with the earlier leaflets, all participating entities validated the guide’s content prior to publication.

Direct access to the Guide for health professionals: https://scientiasalut.gencat.cat/handle/11351/9636

In March 2025, we designed a poster (22) that we sent to 2,000 religious entities in Catalonia, along with a letter signed by the directors of OCATT and DGRA. In that letter, we explained the project carried out few years ago, provided information about organ and tissue donation and offered the possibility of giving an informative talk to the centres themselves about the transplant system.

### Evaluation of the percentage of family consent to donation

**Figure 2:**
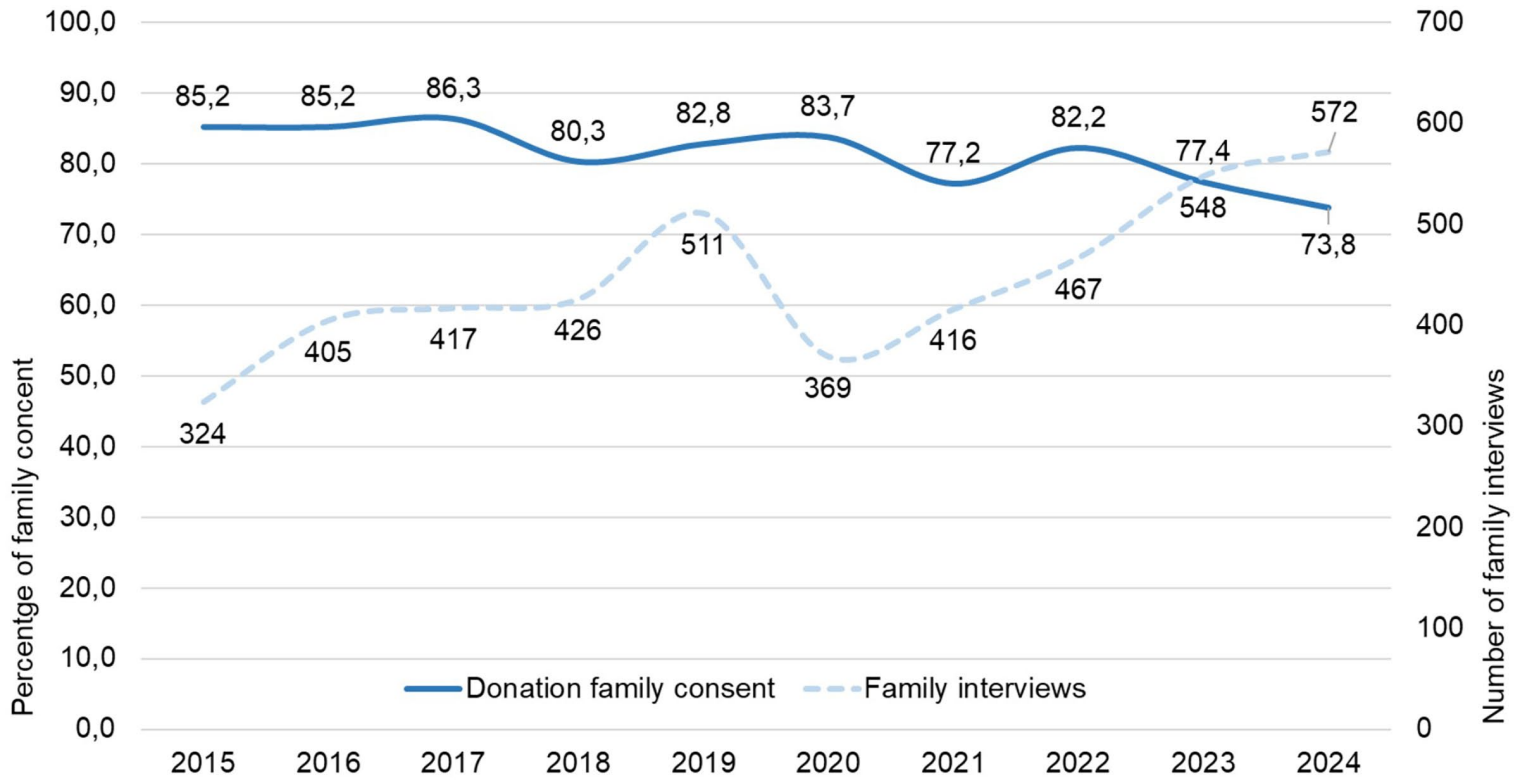
Evolution of the donation consent percentage and family interviews in Catalonia over the last 10 years (2015–2024).

The medium percentage of consent for organ donation in Catalonia over the past 10 years (2015–2024) stands at 81.8% in contrast with 18.2% of families interviewed refused organ donation from a family member. The evolution of this acceptance percentage over the past 10 years (Figure 2) reduced from 85.2% in 2015 to 73.8% in 2024. Comparing this percentage over 5-year periods, 2015–2019 and 2020–2024, which roughly coincide with the pre- and post-Project period, in addition to the COVID-19 pandemic, family acceptance showed a 5-point decrease: 83.8% in 2015–2019 vs. 78.4% in 2020–2024 (Table 1), with statistically significant difference (*p* < 0.001).

**Table 1:** Percentage of family consent to donation (and number of family consent and family interviews) in Catalonia by origin, 2015–2024.

Origen	2015	2016	2017	2018	2019	2015–2019	2020	2021	2022	2023	2024	2020–2024	2015–2024
Spain	86.6 (252/291)	87.3 (322/369)	87.9 (335/381)	81.9 (304/371)	84.3 (382/453)	85.5 (1,595/1865)	83.4 (281/453)	77.8 (301/453)	83.3 (359/453)	78.6 (382/453)	75.0 (382/509)	79.3 (1,705/2150)	82.2 (3,300/4015)
Western Europe	66.7 (8/12)	69.2 (9/13)	75.0 (6/8)	77.8 (14/18)	82.4 (14/17)	75.0 (51/68)	93.3 (14/15)	100.0 (4/4)	92.3 (12/13)	81.8 (9/11)	75.0 (9/12)	87.3 (48/55)	80.5 (99/123)
Eastern Europe	75.0 (3/4)	75.0 (3/4)	100.0 (4/4)	75.0 (3/4)	87.5 (7/8)	83.3 (20/24)	100.0 (1/1)	75.0 (3/4)	100.0 (1/1)	87.5 (7/8)	62.5 (5/8)	73.9 (17/23)	78.7 (37/47)
Middle Est	0.0 (0/1)	– (0/0)	– (0/0)	0.0 (0/2)	0.0 (0/1)	0.0 (0/4)	0.0 (0/1)	– (0/0)	100.0 (1/1)	75.0 (3/4)	20.0 (1/5)	45.5 (5/11)	33.3 (5/15)
Asia	60.0 (3/5)	60.0 (3/5)	0.0 (0/4)	25.0 (1/4)	50.0 (4/8)	42.3 (11/26)	– (0/0)	33.3 (1/3)	0.0 (0/1)	50.0 (2/4)	60.0 (3/5)	46.2 (6/13)	43.6 (17/39)
North Africa	50.0 (1/2)	0.0 (0/4)	0.0 (0/1)	40.0 (2/5)	25.0 (1/4)	25.0 (4/16)	50.0 (1/2)	50.0 (1/2)	0.0 (0/3)	33.3 (3/9)	25.0 (1/4)	30.0 (6/20)	27.8 (10/36)
Sub-Saharan Africa	100.0 (1/1)	50.0 (1/2)	60.0 (3/5)	100.0 (3/3)	50.0 (1/2)	69.2 (9/13)	100.0 (2/2)	100.0 (1/1)	0.0 (0/1)	0.0 (0/1)	0.0 (0/1)	37.1 (3/8)	57.1 (12/21)
North America	– (0/0)	100.0 (1/1)	– (0/0)	100.0 (1/1)	– (0/0)	100.0 (2/2)	**–** (0/0)	**–** (0/0)	100.0 (1/1)	100.0 (2/2)	– (0/0)	100.0 (3/3)	100.0 (5/5)
Latin America	100.0 (8/8)	85.7 (6/7)	85.7 (12/14)	77.8 (14/18)	77.8 (14/18)	83.1 (54/65)	90.9 (10/11)	66.7 (10/15)	69.2 (9/13)	66.7 (16/24)	84.0 (21/25)	75.0 (66/88)	78.4 (120/153)
Oceania	– (0/0)	– (0/0)	– (0/0)	– (0/0)	– (0/1)	– (0/0)	– (0/0)	– (0/0)	100.0 (1/1)	– (0/0)	– (0/1)	50.0 (1/2)	50.0 (1/2)
**Total**	**85.2** (276/324)	**85.2** (345/405)	**86.3** (360/417)	**80.3** (342/426)	**82.8** (423/511)	**83.8** (1746/2083)	**83.7** (309/369)	**77.2** (321/416)	**82.2** (384/467)	**77.4** (424/548)	**73.8** (422/572)	**78.4** (1,860/2373)	**80.9** (3,606/4456)

Family consent to donation percentage.

(Number of family consent to donation/Number of family interviews).

Comparison by periods (2015–2019 vs. 2020–2024) *p* < 0.001.

Comparison by origin *p* < 0.01 (Oceania and North America were excluded due to the low number of cases. Middle Est and North Africa were grouped for this analysis).

In light gray, the grouped results for both 5-year periods. In dark gray, the grouped results for the entire 10-year study period.

In bold, results regardless of the person’s origin.

Likewise, we observed an increase in the number of interviews conducted (Figure 2) over the years, with figures exceeding 500 interviews in 2019, 2023 and 2024. If in the first period (2015–2019), 2,083 family interviews were conducted in Catalonia, while in the second (2020–2024), there were 2,373, 13.9% more, despite the 2 years of strong impact of COVID-19 pandemic in organ donation rates (Table 1).

**Figure 3:**
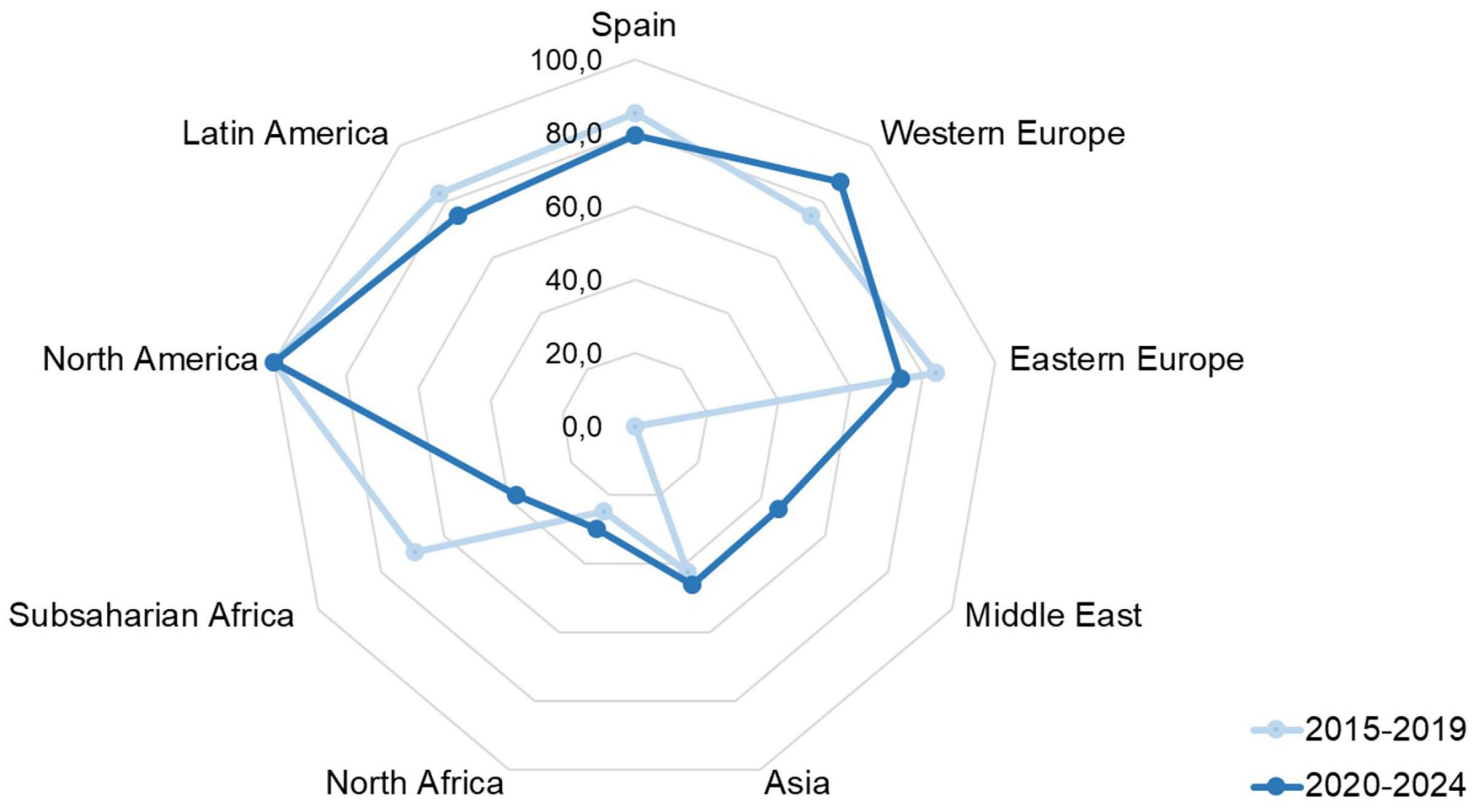
Consent to organ donation in Catalonia by origin of potential donors in both periods grouped, 2015–2019 and 2020–2024.

We observed statistically significant differences (*p* < 0.01) in family consent percentage for donation according to the origin of potential donors (Table 1); people born in Europe, America, or Oceania have higher consent rates than those born in Asia or Africa. When we compared this percentage across the two study periods, we saw significant variations based on origin (Figure 3). Therefore, consent rate decreased among people born in Spain, Central and South America, Eastern Europe, and sub-Saharan Africa, while it increased among those born in Europe, North Africa, Asia, and the Middle East.

## Discussion

No major religion explicitly prohibits organ and tissue donation. The 70 cultural and religious representatives who participated in the Barcelona Reflection Cycle echoed this view. However, while most faiths do not oppose donation, they vary widely in how they approach it (24). Some actively support it—such as the Catholic Church (25)—while others express ambivalence (26) due to tensions between ethical values and specific rites or beliefs around death. Many leaders cited the lack of explicit references in sacred texts as a barrier to consensus and emphasized the need for high-level guidance. In some cases, internal diversity within a single faith further complicates the adoption of a unified stance (27). For others, donation remains a matter of individual conscience.

Our experience, in line with other studies (28, 29), confirms that accurate information fosters positive attitudes toward donation. At the outset of the Barcelona project, we hypothesized that cultural reluctance stemmed more from misinformation and distrust than from a lack of solidarity. Engaging directly with 70 religious and community leaders allowed us to challenge misconceptions, build mutual understanding, and lay the groundwork for future collaboration. The UK’s example (30, 31) of integrating religious voices into donation systems shows this model’s potential, which we have started to replicate in Catalonia. These partnerships have already enabled coordinated support during family interviews, leading us to propose the role of a community mediator within transplant teams. In diverse urban settings, this figure could provide culturally sensitive information and spiritual support during critical moments.

Trust and credibility underpin the Spanish donation system. Addressing myths and improving public knowledge—especially within specific cultural groups—reinforces this foundation. In societies where myths and end-of-life taboos persist, donation values like altruism, solidarity, mercy, and compassion become powerful tools (32). These values, common across many faiths and central to the Barcelona Reflection Cycle, helped build bridges and raise awareness. All religious leaders involved underscored their relevance in promoting donation for the collective good.

Health Professionals—especially the Transplant Coordinator—play a key role in respecting families’ cultural and spiritual needs. In this sense, our experience in effectively communicating bad news and building a necessary bond of trust, compassion, and empathy with patients’ families suggests that, their understanding of organ donation as an integral part of end-of-life care, begins the moment a potential donor with catastrophic brain or cardiac injury—and their family—arrive at the hospital. Training initiatives focused on religious and cultural diversity are essential to improve these interactions, particularly when healthcare teams are themselves multicultural. Therefore, informative, educative or training initiatives aimed at understanding the diversity of cultures and religious beliefs present in our societies (33) are essential elements to favour understanding and communication, especially when multicultural healthcare teams are incorporated in the donation process (34, 35).

Families also struggle with complex medical and ethical concepts—such as brain death, futility, or withdrawal of treatment—especially in donation after circulatory death. Language barriers and time constraints add further pressure. In these cases, support from religious or cultural mediators can improve communication and facilitate consent. This approach is gaining traction in other countries (36) and has started in Catalonia thanks to connections made through this project.

In real-life practice, we can see all these aspects reflected in the data on family consent to donation. This percentage—or conversely, the rate of family refusals—serves as one of the core indicators of our transplant system. In this study, we tracked its evolution over the past 10 years, analysing it year by year and grouping the data into two five-year periods that roughly align with the timeline before and after the Barcelona Reflection Cycle. Each year, the percentage fluctuates, showing a slight downward trend that may indicate a gradual decline in family agreement to donation. This becomes especially clear when we compare the two five-year periods, which reveal a decrease of 5% points.

Although the results might suggest the Barcelona project did not raise awareness among the Catalan population, consent rates actually declined among people born in Spain, Latin America, and sub-Saharan Africa, but increased among groups from Europe, Asia, North Africa, and especially the Middle East, despite their smaller donor pool. These trends confirm the project’s original aim—understanding the influence of diverse religions beyond Catholicism—remains relevant to improving outcomes in specific populations.

Cross-country comparisons remain difficult, but studies from Italy (18, 37), the UK (38), and Norway (39) report lower acceptance among non-European communities than what we observed. Spain’s consistently high family consent rates—higher than in most countries (40)—suggest that integration into a strong donation system (41) may promote greater acceptance, even among migrant communities. For example, British nationals residing in Spain show higher consent rates than those in the UK (42).

An important issue is the decline in donation consent among Spanish families, likely linked to broader social changes—such as rising individualism and a weaker sense of the common good (43)—as well as factors within the hospital setting, including interview timing, communication, and team functioning. A thorough study of family interviews and refusals is therefore essential.

Beyond the knowledge we have gained and the educational materials we have developed—both for families and for professionals, which always bring value—our contact with religious leaders has proved especially positive. These leaders have directly participated in some family interviews, regardless of their outcome, and now offer us a close and accessible resource that helps us better support and connect with families from different cultural backgrounds. This progress stems directly from the workshops held as part of the Barcelona project, which gave us the chance to create and share a two-way space for knowledge: a moment of meaningful, grateful, and productive dialogue between two spheres that, until now, had rarely interacted.

The relationship between organ donation and the cultural or religious world does not end there. Over the next few months, we have two main, complementary activities planned. First, we will organize informational talks given by medical students to youth groups from different cultures. This activity has two clear advantages: engaging and raising awareness among young people, and strengthening the understanding and integration of youth communities from diverse cultures and religions. In addition, we want to create a collection of videos with testimonials from recipients of different cultures and ages.

Besides everything we have accomplished so far, another success of the project has been to integrate the DGRA as a current and future asset for the transplant system. Proof of this is that when a transplant coordinator needs a religious mediator to speak with a family, regardless of their beliefs, we can provide him one.

### Limitations

A main limitation is the partial participation of religious groups; some chose not to engage, introducing potential selection bias, though they were a minority. Collaboration with community leaders and educational tools does not guarantee awareness or behavioural change. This initiative is only a first step, highlighting the need for sustained partnerships between transplant programs and faith-based organizations.

Another limitation is the accuracy and consistency of consent data. Reporting of family interviews has become more rigorous over time, affecting comparability. Moreover, the low number of potential donors born outside of Spain, especially in certain groups based on their origin (Table 1), makes it difficult to obtain statistically significant results, since small variations can disproportionately affect the percentages. Therefore, the objective of the analysis was only to describe the situation in Catalonia over the last few years.

**Figure 4:**
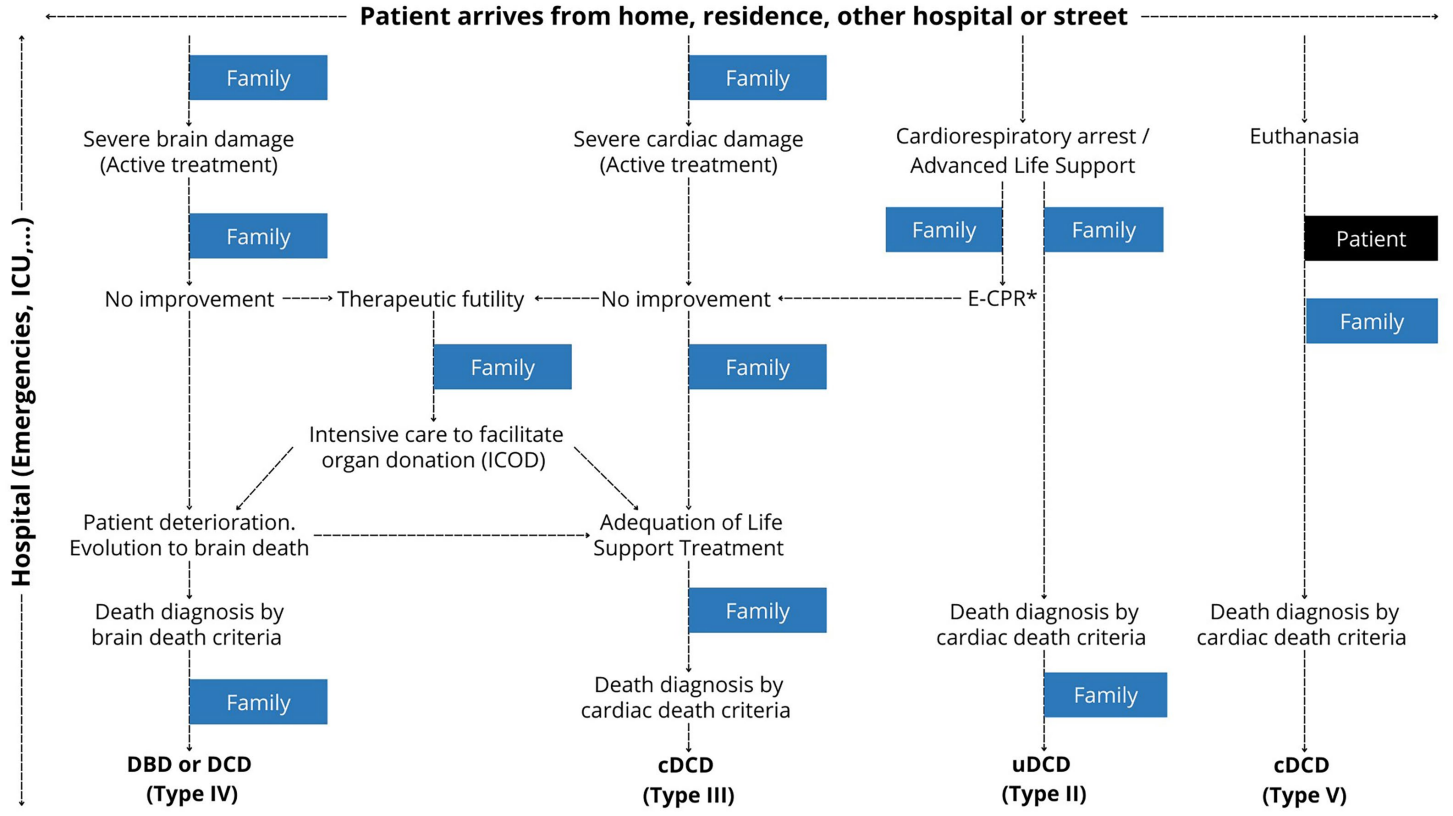
New scenarios for interaction with a donor family. The donation process has evolved with the rise of donation after circulatory death and expanded donor sources. Family interviews, once conducted exclusively by transplant coordinators as the final step, now sometimes occur earlier and may involve other professionals. DBD, donor after brain death; DCD, docor after cardiac death (u: uncontroled. c: controled); E-CPR, extracorporeal cardiopulmonary resuscitation.

Finally, since 2014, the donation process has evolved—with donation after circulatory death and expanded donor sources—while interviews, once conducted only at the end by TC (8), may now occur earlier and involve other professionals (Figure 4). These changes likely influence consent rate comparisons (44). In this sense, the Spanish model also integrates organ donation as part of end-of-life care through the early identification and referral of potential donors from the emergency department to the intensive care unit. Both in patients receiving active treatment who deteriorate and progress to brain death and in those for whom treatment is considered futile and adequacy of life-sustaining treatment is contemplated, the transplant coordination team works with the clinical staff to inform and support families and to propose organ donation. A proactive and coordinated approach between transplant coordination teams and family care allows support to be provided throughout the entire care process, facilitates understanding of the severity of the situation, promotes appropriate grieving, and addresses the possibility of organ donation in a respectful manner. Unfortunately, this level of involvement does not occur in all cases.

## Conclusion

The Barcelona project launched 6 years ago, pioneered collaboration between healthcare and religious communities. In today’s diverse society, it fostered new partnerships and strengthened spiritual care. Its educational tools now help transplant coordinators connect more effectively with families of different cultural and religious backgrounds. As Dra. Lena de Bottom noted at the end of Judaism workshop, *“Émile Durkheim said that a society that does not know itself needs connections, and in this case, donation and trust in the transplant system are one of them.”*

## Data Availability

The raw data supporting the conclusions of this article will be made available by the authors, without undue reservation.
