# Supplementary material for: Organ donation: a cultural and religious vision. The Barcelona project
Source: Front Public Health. 2026 Apr 8;14:1773397. doi: 10.3389/fpubh.2026.1773397 (PMC13099779; doi:10.3389/fpubh.2026.1773397)
Supplement: Supplementary file 1 [file Supplementary_file_1.pdf]

Donation and religious diversity

A cultural and religious vision of the organ and tissue donation

Why?

Trust and credibility of the society in donation is a key factor for Spanish model success. In 2019 a “Donation and religions project” was conceived as a multidisciplinary approach to discuss donation and transplantation concepts with religious leaders from Catalonia, with the aim to improve knowledge about religious or cultural aspects that could encourage dialogue and reduce refusal for donation.

The Barcelona Project

The project took place at the Palau Macaya in Barcelona between 2018 and 2019, consisting of 7 in-person events: 2 open conferences and 5 closed seminars. Each workshop focused on a particular faith or group of religions: Catholicism, rest of Christianity, Islam, Judaism, and Asian religions.

Palau Macaya de Barcelona
(“la Caixa” Foundation)

OCATT
Catalan Transplants Organitzation
(Catalan Health System)

G.D. Religious Affairs
(Catalan Goverment)

10 expert speakers
(from OCATT, Catalan hospitals, NHS, ONT and WHO)

Participate in the **2 conferences**: opening and clousure

70 leaders or representatives
from 70 cultural and religious organizations of Catalonia

16 experts moderators
(11 transplants coordinaros of Catalan Hospitals and 5 members of Religious Diversity Board of Catalonia)

Participate in any of the **5 seminars**:

Catholicism
Non-Catholic Christianism
Islam
Judaism
Asian religions

Main results

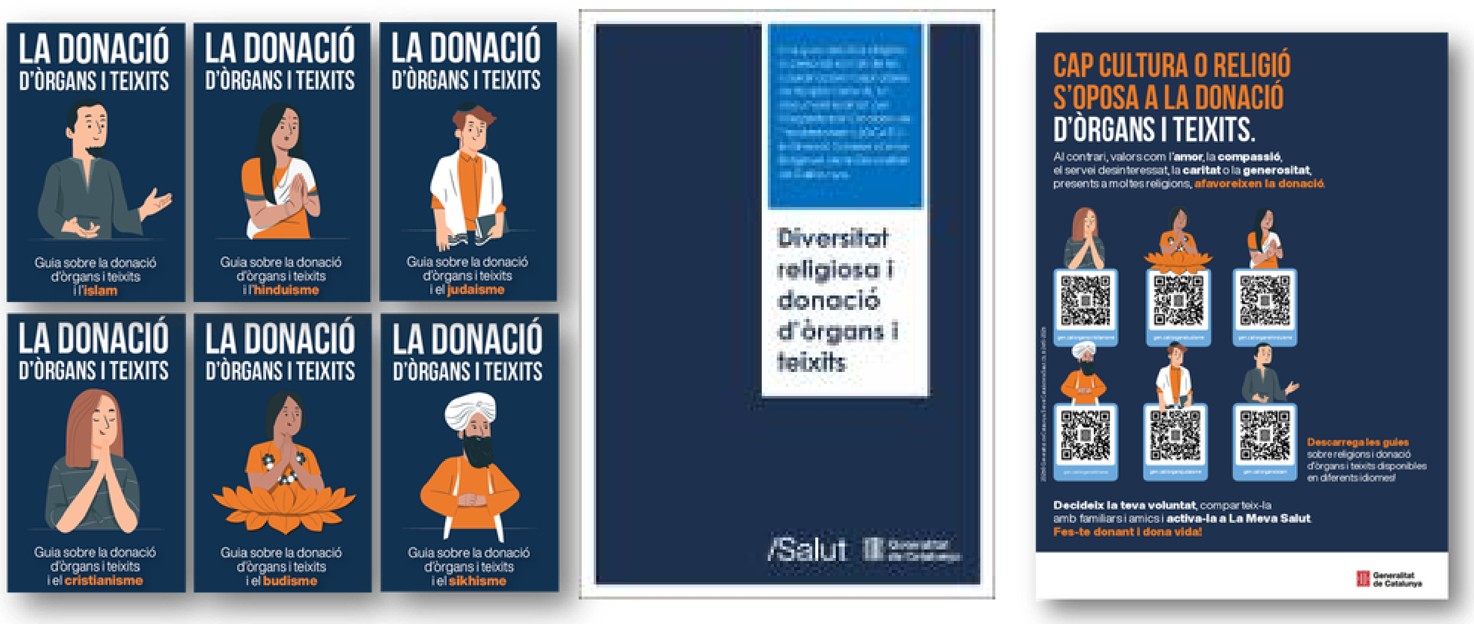

- Knowledge and information
- **6 leaflets** for general population
- **1 Guide** of religion diversity and donation for professionals
- **1 poster for** health and religious centres
- Useful local contacts of diferent faiths

Family consent to donation in Catalonia

Family interview is a mandatory part of the donation process.
The percentatge of family donation consent or, in the opposite, the percentatge of family refusals is a key indicator of the donation process.

Figure. Percentage evolution of the family consent to donation in Catalonia, 2015-2024

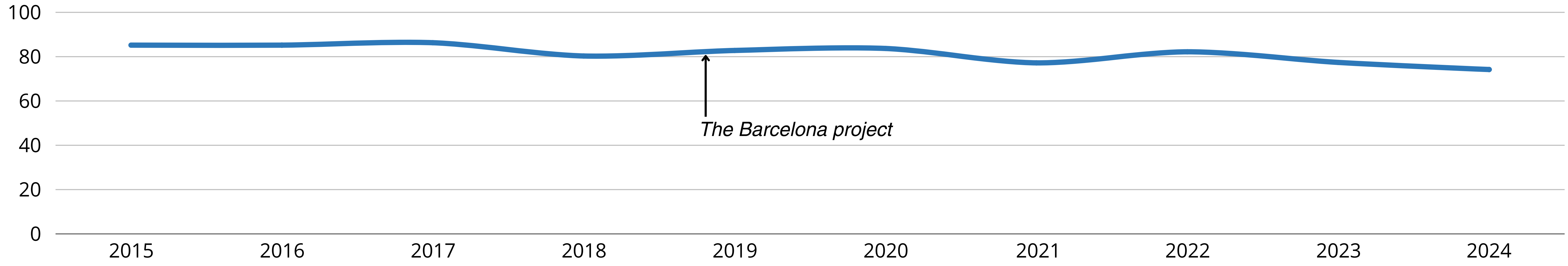

Figure. Percentatge of family consent to donation in Catalonia by origin and period, 2015-2029 and 2020-2024.

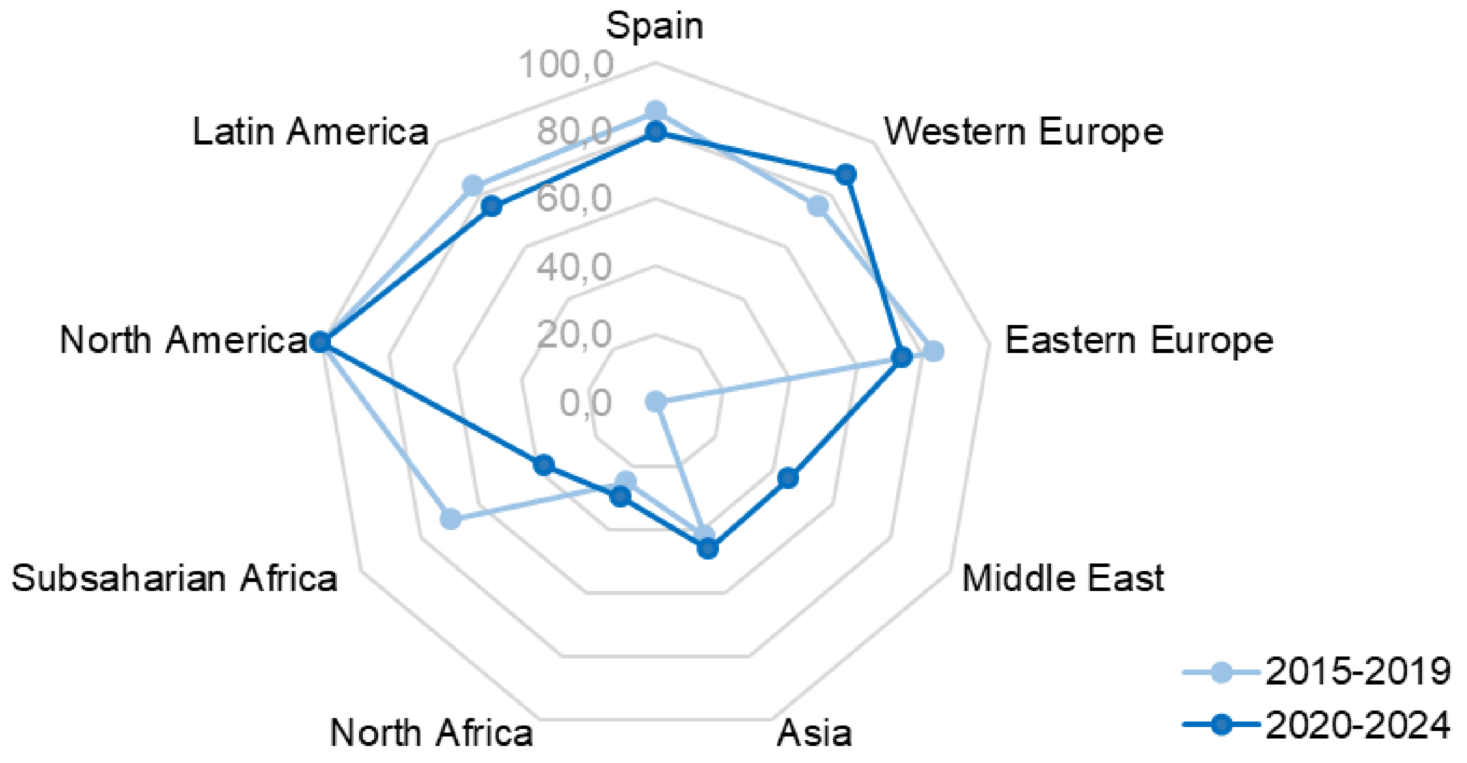

Conclusion
The Barcelona project was a pioneering initiative within our healthcare system and gained broad support from the religious community. In an increasingly diverse society, fostering new forms of collaboration and strengthening spiritual care in clinical settings has become essential. The educational materials and relationships established through the project now help transplant coordinators build closer, more informed connections with families from different cultural and religious backgrounds.

Organització Catalana de Trasplantaments (**OCATT**). 2026. Contacte: ocatt@catsalut.cat
